# Prevention of infectious complications after transrectal ultrasound-guided prostate biopsy: comparison of povidone-iodine, chlorhexidine, and formalin disinfection

**DOI:** 10.1007/s00345-025-05498-4

**Published:** 2025-02-07

**Authors:** Mert Başaranoğlu, Ali Nebioğlu, Murat Bozlu, Ali Gökçe, Erdem Akbay

**Affiliations:** 1 Department of Urology, Mersin University Faculty of Medicine, Ciftlikkoy Campus, Yenişehir, Mersin, Turkey; 2 Department of Urology, Mersin City Training and Research Hospital, Mersin, Turkey; 3 Department of Obstetrics and Gynecology, Ankara University Medical School, Ankara, Turkey

**Keywords:** Chlorhexidine, Formalin, Infectious complications, Povidone-iodine, Prostate biopsy

## Abstract

**Purpose:**

We aimed to compare the efficacy of three different antiseptic methods to determine the most effective prophylactic approach to prevent infectious complications after transrectal ultrasound-guided prostate biopsy (TRUS-PB). The methods evaluated were transrectal povidone-iodine injection (TRPI), biopsy needle disinfection with chlorhexidine, and biopsy needle disinfection with formalin.

**Methods:**

Between January 2018 and January 2023, 904 patients who underwent TRUS-PB were retrospectively analyzed. All patients had prophylactic antibiotic use and negative urine/rectal culture results. Patients were divided into three groups according to the antiseptic protocol: Group 1 (*n* = 245) received only TRPI injection into the rectum before biopsy, Group 2 (*n* = 295) received only chlorhexidine needle disinfection before biopsy, and Group 3 (*n* = 364) received only formalin needle disinfection before biopsy. The biopsy needles used in our clinic are not single-use and are used on other patients after sterilization. The primary endpoint was the incidence of infectious complications within 30 days post-procedure. Continuous variables were analyzed using the Mann-Whitney U test, while categorical variables were analyzed using the Chi-square test, and post-hoc analysis was applied for pairwise comparisons between groups. Univariate and multivariate logistic regression analysis was performed to evaluate factors associated with postoperative infection.

**Results:**

The overall infection rate was 20.4%. Infection rates were 4.5% in the TRPI group, 16.6% in the chlorhexidine group, and 34.1% in the formalin group (*p* < 0.001). The TRPI group showed significantly lower rates of all infectious complications compared to other groups. Disinfection of biopsy needles with chlorhexidine was found to be significantly more effective in preventing infectious complications compared to disinfection with formalin (*p* < 0.001).

**Conclusion:**

TRPI injection before TRUS-PB appears to be more effective in preventing post-biopsy infectious complications compared to needle disinfection with chlorhexidine or formalin. This method may be considered as a preferred antiseptic approach for TRUS-PB procedures.

## Introduction

Prostate cancer (PCa) is the second most common cancer among men worldwide and the fifth leading cause of cancer-related death [1]. The discovery of prostate-specific antigen (PSA) has led to significant advances in the diagnosis of PCa, and it remains the most important and widely used biomarker in the diagnostic process today [2]. The widespread use of PSA in clinical practice has also contributed to a substantial increase in PCa incidence. The most significant parameters that maintain validity in the preliminary diagnosis of PCa are digital rectal examination (DRE) and PSA evaluations. Multiparametric prostate magnetic resonance imaging (MRI) has become an important step in the PCa diagnostic algorithm after PSA elevation. Randomized controlled trials have shown that the use of MRI optimizes the detection of clinically significant prostate cancers while reducing the number of unnecessary biopsies. In the evaluation performed using the prostate imaging reporting & data system (PI-RADS) scoring system, especially in patients with PSA > 3 ng/mL, the inclusion of MRI in the diagnostic algorithm prevents overdiagnosis of low-risk cancers and enables the detection of clinically significant cancers [3]. In cases of elevated PSA levels and/or pathological findings on DRE, in the presence of MRI findings suspicious for cancer histopathological examination is required for a definitive diagnosis of PCa. Transperineal or transrectal prostate biopsy procedures are performed for this histopathological examination. Transrectal ultrasound-guided prostate biopsy (TRUS-PB) remains a widely used standard procedure in the diagnostic evaluation of patients with suspected PCa, with over one million applications performed annually worldwide [4]. In this biopsy procedure, prostate tissue is accessed through the rectum, which is rich in blood vessels and has a dense bacterial flora. Consequently, postoperative urological infections are primarily attributed to contamination and inoculation from rectal flora. Although the TRUS-PB procedure is generally well tolerated and considered safe by patients, it can lead to infectious complications such as urinary tract infections, epididymo-orchitis, prostatitis, and rarely, life-threatening urosepsis [5, 6]. The incidence of dysuria is 14%, and the incidence of urinary tract infections is 10%. More severe complications requiring hospitalization, such as sepsis and septic shock, have incidences of 5.7% and 0.45%, respectively, with a reported mortality rate of 0.2% associated with these conditions [6].

Both the European Association of Urology (EAU) and the American Urological Association (AUA) guidelines recommend appropriate antibiotic prophylaxis before TRUS-PB to prevent infectious complications [7, 8, 9]. Ciprofloxacin, a fluoroquinolone, is frequently preferred for prophylaxis before TRUS-PB due to its good diffusion into the prostate parenchyma and high activity against intestinal flora [10, 11]. However, studies have shown an increasing resistance to quinolones among microorganisms, resulting in significant increases in hospitalization rates following prostate biopsy [12].

Numerous studies have been conducted to reduce infectious complications after TRUS-PB. Some of these include obtaining anal swab cultures before the procedure and applying antibiotic prophylaxis based on the culture results, cleaning the biopsy needle tip with formalin or chlorhexidine, and favoring transperineal approaches over transrectal approaches in current practices [13, 14]. However, studies comparing transperineal and transrectal prostate biopsies have not found significant superiority in diagnosing PCa [15], although transperineal biopsies have been shown to lead to significantly lower rates of infectious complications compared to transrectal biopsies. Despite the lower infectious complication rates associated with transperineal biopsies, they are not as widely used due to disadvantages such as the requirement for longer procedural times, and the need for specialized equipment, though they are presented as a good alternative to TRUS-PB due to their lower rates of serious complications [16].

Current prophylactic methods typically involve a combination of local antiseptics and systemic antibiotics. Povidone-iodine and chlorhexidine are commonly used antiseptics, while fluoroquinolones, especially ciprofloxacin, have been the mainstay of antibiotic prophylaxis. However, rising rates of antibiotic resistance have led to the investigation of alternative antibiotics, such as cefpodoxime, a third-generation cephalosporin [17, 18]. Despite these preventive measures, the optimal prophylactic regimen remains a topic of debate. The efficacy of different antiseptic-antibiotic combinations in reducing infection rates after TRUS-PB has not been thoroughly compared in large-scale studies. Additionally, the impact of local bacterial resistance patterns on the effectiveness of these prophylactic strategies is not well understood.

This study aims to fill these knowledge gaps by comparing the effectiveness of different antiseptics, such as transrectal povidone-iodine injection (TRPI), chlorhexidine, and formalin in the disinfection of the biopsy needle tip, in reducing infection rates after TRUS-PB. By analyzing a large patient cohort, we aim to provide evidence-based recommendations for optimizing prophylaxis protocols in TRUS-PB procedures, potentially reducing infection rates and improving patient outcomes.

## Materials and methods

### Data sources and study population

This retrospective study analyzed 904 TRUS-PB procedures performed between January 2018 and January 2023. Each biopsy procedure was analyzed as an independent event. The records of male patients aged 45–75 who met the inclusion criteria and underwent TRUS-PB at Mersin University Urology Clinic were analyzed. Common biopsy indication criteria for all patients were defined as pathological findings on DRE (regardless of PSA levels) and/or PSA levels > 4 ng/ml. Prior to the biopsy, urine cultures and rectal swab cultures were obtained from all patients. Only patients who underwent systematic 12 core prostate biopsy were included in our study. Patients with allergies to cefpodoxime, chlorhexidine, phosphate, or povidone-iodine, those with positive cultures in urine or rectal swabs, hematological diseases, uncontrolled diabetes, permanent urethral catheters, severe hemorrhoids, thyroid dysfunction, or those receiving radioactive iodine therapy were excluded from the study. Ethical approval for the study was obtained from the Local Ethics Committee of Mersin University Faculty of Medicine with decision number 2023/695 dated October 18, 2023. Informed consent was obtained from all patients prior to the procedure, including an explanation of the procedure and potential risks.

All patients received prophylactic cefpodoxime (200 mg orally, twice daily) for 10 days starting the day before the procedure. Additionally, all patients underwent a cleansing enema containing monobasic and dibasic sodium phosphate approximately twelve hours and two hours prior to the procedure.

The study included 245 patients who only received TRPI, 295 patients who had their biopsy needles disinfected only with chlorhexidine, and 364 patients who had their biopsy needles disinfected only with formalin. The biopsy needles used in our clinic are not single-use and are used on other patients after sterilization. For TRPI, a 10% povidone-iodine solution was injected into the rectum using a 50 cc gavage syringe 15 min prior to the procedure. Needle disinfection was performed before each puncture using either chlorhexidine (4% solution, 10-minute immersion) or formalin (10% solution, 10-minute immersion).

All patients were positioned in the left lateral decubitus position and received periprostatic block with bupivacaine, administered by the same urologist under transrectal ultrasound guidance (General Electric Logiq 500 Pro Series Ultrasound device), after which 12-core prostate biopsies were obtained using an automatic biopsy gun and an 18 G Tru-Cut biopsy needle (Geotek Medical, Turkey). Patients were followed for 30 days post-biopsy and were instructed to report to the emergency department for any signs of infection (fever, dysuria, increased urinary frequency, or perineal pain). Infectious complication criteria were defined as symptoms of urinary tract infection (such as increased frequency, urgency, dysuria, etc.) plus a body temperature > 37.8 °C. Sepsis was defined by the presence of symptoms of systemic inflammatory response along with positive cultures indicating infection. All patients presenting with infectious complications were hospitalized for further evaluation and treatment.

### Statistical analysis

Sample size was calculated using G*Power software (version 3.1.9.7) with an effect size of 0.25, alpha error of 0.05, and power of 0.90 for three groups, yielding a minimum required sample size of 207 patients per group. To account for potential dropouts and missing data, we included 904 patients.

Statistical analyses were conducted using IBM SPSS Statistics for Windows, Version 25.0 (IBM Corp., Armonk, NY, USA). The normality of data distribution was assessed using the Kolmogorov-Smirnov test. Descriptive statistics for continuous variables were expressed as median (minimum-maximum), while categorical variables were expressed as counts and percentages. Mann-Whitney U test was used for comparisons of continuous variables, and the Chi-square test was used for categorical variables. Post-hoc analysis with Bonferroni correction was applied for evaluating differences among the three groups. Demographic and anthropometric parameters (age, body weight, height, body mass index), comorbidity status, hereditary factors, tobacco use history and clinical data of the patients were evaluated with multivariate analysis methodology. Univariate logistic regression analysis was performed to evaluate factors associated with postoperative infection. A p-value < 0.05 was considered statistically significant.

## Results

A total of 904 male patients who met the inclusion criteria were analyzed in this study. The demographic and anthropometric parameters of the patients, including age, body weight, height, body mass index (BMI), comorbid conditions, hereditary factors, and smoking history, were evaluated using a multivariate analysis methodology, revealing no statistically significant differences between the groups (*p* > 0.05). The distribution of infectious complications was as follows: urinary tract infection was observed in 170 patients (18.8%), and sepsis was identified in 14 patients (1.54%). All descriptive statistical data are summarized in Table 1.

**Table 1:** Distribution of infectious complications post-TRUS-PB

Category	*n*	(%)
**Total patients**	904	100
**No infections**	720	79.6
**Infectious complications**	184	20.4
Urinary tract infection	170	18.8
Sepsis	14	1.54

The median age of patients in the non-infectious group was 61 years (49–72), while the median age in the infectious group was 62 years (45–75) (*p* = 0.210). The median BMI in the non-infectious group was 25.55 kg/m² (18.5–29.4), whereas in the infectious group, it was 25.51 kg/m² (19.8–29.4) (*p* = 0.682). Smoking rates were evaluated; 209 patients (29.03%) in the non-infectious group were smokers, while 50 patients (27.17%) in the infectious group smoked (*p* = 0.685). The median PSA level in the non-infectious group was 14.14 ng/mL (4.0-24.95), compared to 15.38 ng/mL (4.04–24.99) in the infectious group (*p* = 0.398). No statistically significant differences were found between the two groups regarding age, PSA levels, BMI, smoking status, or comorbidities. All descriptive statistical data are summarized in Table 2.

**Table 2:** Statistical comparison of patient groups with and without post-biopsy infection in terms of age, BMI, smoking status, and prostate specific antigen values

	Post-biopsy infection positive (*n* = 184)	Post-biopsy infection negative (*n* = 720)	*p*
**Age**,** median (min-max)**	62 (45–75)	61 (49–72)	0.210
**PSA**,** median (min-max)**	15.38 (4.04–24.99)	14.14 (4–24.95)	0.398
**BMI**,** median (min-max)**	25.51 (19.8–29.4)	25.55 (18.5–29.4)	0.682
**Smoking**,** n (%)**	50 (27.17)	209 (29.03)	0.685
**Comorbidity**			0.735
*None*,* n (%)*	25 (13.59)	104 (12.44)	
*Diabetes mellitus*,* n (%)*	25 (13.58)	82 (11.39)	
*Hypertension*,* n (%)*	22 (11.96)	107 (14.86)	
*Coronary artery disease*,* n (%)*	18 (9.78)	87 (12.08)	
*Diabetes mellitus and Hypertension*,* n (%)*	21 (11.41)	90 (12.50)	
*Hypertension and Coronary artery disease*,* n (%)*	29 (15.76)	84 (11.67)	
*Diabetes mellitus and Coronary artery disease*,* n (%)*	24 (13.04)	89 (12.36)	
*Diabetes mellitus*,* Coronary artery disease and Hypertension*,* n (%)*	20 (10.87)	77 (10.69)	

PSA: prostate specific antigen, BMI: body mass index

The rates of infectious complications in the TRPI group were found to be 4.5% (11/245), in the chlorhexidine group 16.6% (49/295), and in the formalin group 34.1% (124/364). TRPI injection was statistically significantly more effective in preventing infectious complications than the other two methods (*p* < 0.001). Disinfection of biopsy needles with chlorhexidine was found to be significantly more effective in preventing infectious complications compared to disinfection with formalin (*p* < 0.001). All statistical data are summarized in Table 3.

**Table 3:** Comparison of infectious complication rates and statistical analysis results among transrectal prostate biopsy with local anesthesia, chlorhexidine, and formalin groups

Groups	Post-biopsy infection positive (*n* = 184)	Post-biopsy infection negative (*n* = 720)	*p*
Povidone iodine, *n* (%)	11 (4.5%)	234 (95.5%)	**< 0.001**
Chlorhexidine, *n* (%)	49 (16.6%)	246 (83.4%)	
Formalin, *n* (%)	124 (34.1%)	240 (65.9%)	

### Univariate and multivariate analysis

The multivariate logistic regression analysis revealed the following results for potential confounding factors: age (OR = 1.013, 95% CI [0.991–1.037], *p* = 0.250), PSA levels (OR = 1.012, 95% CI [0.985–1.039], *p* = 0.388), BMI (OR = 1.02, 95% CI [0.937–1.11], *p* = 0.648), smoking status (OR = 0.912, 95% CI [0.635–1.311], *p* = 0.620), and presence of comorbidities (OR = 1.074, 95% CI [0.671–1.718], *p* = 0.767). When using povidone-iodine as a reference variable, chlorhexidine showed significantly higher risk of postoperative infections (OR = 4.23, 95% CI [2.151–8.347], *p* < 0.001), and formalin exhibited an even stronger association with increased infection risk (OR = 10.991, 95% CI [5.782–20.893], *p* < 0.001). These comprehensive results are presented in Table 4.

**Table 4:** Univariate and multivariate regression analyses were performed to evaluate factors associated with postoperative infection

	Univariate OR (95% CI)	*P*	Multivariate OR (95% CI)	*P*
**Age**	1.013 (0.991–1.037)	0.250	-	> 0.05
**PSA**	1.012 (0.985–1.039)	0.388	-	
**BMI**	1.02 (0.937–1.11)	0.648	-	
**Smoking**	0.912 (0.635–1.311)	0.620	-	
**Comorbidity**	1.074 (0.671–1.718)	0.767	-	
**Method (Povidone iodine– reference variable)**				
Chlorhexidine	4.23 (2.151–8.347)	< 0.001	4.23 (2.151–8.347)	**< 0.001**
Formalin	10.991 (5.782–20.893)	< 0.001	10.991 (5.782–20.893)	**< 0.001**

## Discussion

The timely diagnosis and staging of PCa by clinicians are crucial for guiding appropriate treatment choices, preventing under- or overtreatment, and significantly improving patients’ quality of life and survival rates [19]. TRUS-PB is a standard procedure for the diagnostic evaluation of patients with suspected PCa and continues to be widely used in urology clinical practice. However, this invasive procedure can lead to significant costs, morbidity, and, rarely, mortality due to potential complications. Infectious complications can arise post-biopsy, which may result in hospitalization and, in severe cases, serious sepsis or even death. In recent years, the emergence of resistance to fluoroquinolone antibiotics, previously preferred for prophylaxis, has prompted a search for new strategic approaches to combat post-biopsy infectious complications. Alternative strategies include changing prophylactic antibiotic regimens, obtaining preoperative anal swab cultures and administering antibiotic prophylaxis based on culture results, disinfecting biopsy needles with formalin before each biopsy, opting for transperineal biopsy instead of transrectal biopsy, and applying rectal mucosal antisepsis with povidone-iodine or formalin solutions. EAU 2024 Prostate Cancer Guidelines evaluated a meta-analysis of eight studies covering 1.596 patients. As a result of this evaluation, it was determined that infectious complications were significantly reduced in patients who underwent transperineal biopsy compared to transrectal biopsy, and the guideline presented a strong recommendation for the transperineal approach in prostate biopsy [20]. In consideration of emphasizing the significance of our investigated disinfection strategies, cefpodoxime was selected as the prophylactic antimicrobial agent prior to the biopsy procedure, given its broad-spectrum coverage and established efficacy in urological interventions. Studies have shown that lavaging alone is insufficient to prevent infections following TRUS-PB [21]. Similarly, in studies comparing cefpodoxime with ciprofloxacin, significantly lower rates of adverse events and incidence of urinary tract infections were found in the cohort of patients treated with fosfomycin trometamol prophylaxis in the postoperative period, parallel to the results observed with cefpodoxime in prophylaxis before prostate biopsy [22]. For many years, urologists have administered ciprofloxacin as prophylaxis before TRUS-PB; however, the incidence of infectious complications has reached as high as 15%.

This study compares the efficacy of different antiseptic disinfection methods in preventing infections after TRUS-PB and provides valuable insights into optimal antiseptic approaches. The present study demonstrates TRPI injection significantly reduces post-TRUS-PB infectious complications compared to traditional needle disinfection methods. Our findings reveal infection rates of 4.5%, 16.6%, and 34.1% for TRPI, chlorhexidine, and formalin groups, respectively (*p* < 0.001), representing a substantial clinical benefit for TRPI administration. These results align with several pivotal studies in the literature. Park et al. [23] reported that the simple use of suppository type povidone-iodine can effectively prevent infectious complications in TRUS-PB. Ghafoori et al. [24], reported that the use of povidone-iodine as an intrarectal gel can effectively prevent infectious complications in TRUS-PB. Bostancı et al. [25], reported that the combined application of biopsy needle disinfection with formalin solution and rectal cleaning with povidone-iodine is more effective. This consistency across studies underscores the potential of TRPI as a preferred prophylactic approach. However, our findings contrast with Ryu et al. [26], who found no significant benefit with TRPI application. This discrepancy might be attributed to differences in application technique, timing, or concentration of the povidone-iodine solution.

The overall infection rate in our study was found to be 20.4%, which is higher than the reported range in the literature (1–7%). This discrepancy is attributed to our broader definition of infection, which included all symptomatic cases, whereas other studies only considered culture-proven infections.

The strengths of this study include the large sample size and direct comparison of three different prophylaxis regimens. However, the single-center nature and retrospective design of our study limit the generalizability of our findings to larger populations. This study contributed to the existing literature. Further studies are needed to focus on prospective, randomized trials to validate these findings. Additionally, studies that include cost-effectiveness analyses will provide valuable insights for optimizing prophylaxis protocols.

## Conclusion

The study shows that transrectal TRPI injection is more effective in preventing post-biopsy infectious complications compared to needle disinfection with chlorhexidine or formalin. This method demonstrates potential as a preferred antiseptic approach for TRUS-PB procedures. Despite the higher overall infection rate observed in this study compared to existing literature, the findings highlight the superior antimicrobial activity of povidone-iodine in the rectal environment. The study’s strengths include a large sample size and direct comparison of multiple prophylaxis regimens. However, the single-center, retrospective design limits the generalizability of the results. Prospective, randomized trials are needed and include cost-effectiveness analyses to optimize prophylaxis protocols.

## Data Availability

Authors will share data if requested.
